# Clinical and microbiological characterization of *Staphylococcus lugdunensis* isolates obtained from clinical specimens in a hospital in China

**DOI:** 10.1186/1471-2180-12-168

**Published:** 2012-08-06

**Authors:** Chaojun Liu, Dingxia Shen, Jing Guo, Kaifei Wang, Huan Wang, Zhongqiang Yan, Rong Chen, Liyan Ye

**Affiliations:** 1 Department of Clinical Microbiology, General Hospital of The People’s Liberation Army, No. 28 Fu Xing Road, Hai Dian District, Beijing 100853, China; 2 Clinical Laboratory, The 261st Hospital of The People’s Liberation Army, Beijing, China

**Keywords:** *Staphylococcus lugdunensis*, Coagulase-negative staphylococci, Antibiotic resistance, Pulsed-field gel electrophoresis, *mecA*, *ermC*

## Abstract

**Background:**

Several reports have associated *Staphylococcus lugdunensis* with the incidence of severe infection in humans; however, the frequency and prevalence of this microorganism and thus the propensity of its antimicrobial drug resistance is unknown in China. The objective of the current study was to determine the prevalence of *Staphylococcus lugdunensis* among six hundred and seventy non-replicate coagulase negative *Staphylococcus* (CoNS) isolates collected in a 12-month period from clinical specimens in the General Hospital of the People’s Liberation Army in Beijing, China.

**Results:**

Five (0.7%) of the 670 isolates of CoNS were identified as *S. lugdunensis*. Whereas three isolates were resistant to erythromycin, clindamycin, and penicillin and carried the *ermC* gene and a fourth one was resistant to cefoxitin and penicillin and carried the *mecA* gene, one isolate was not resistant to any of the tested antimicrobials. Pulse field gel electrophoretic analysis did not reveal widespread epidemiological diversity of the different isolates.

**Conclusion:**

Hence, even though *S. lugdunensis* may be yet unrecognized and undefined in China, it still might be the infrequent cause of infection and profound multi-drug resistance in the same population.

## Background

*Staphylococcus lugdunensis* is a coagulase-negative staphylococci (CoNS) first described by Freney *et al.*. in 1988 [1] and usually serves as an aetiologic agent of skin and soft tissue infections, mostly in the pelvic and inguinal regions [2]. In recent years, there have been a number of reports on invasive infections of *S. lugdunensis* resulting in destructive clinical outcome [3-6] and this bacterium has become an increasingly important virulent human pathogen [7]. While *S. lugdunensis* can cause infections in multiple tissues, including the skin, soft tissue, bone marrow, peritoneum, central nervous system, blood, endocardium, and joints [8], it primarily infects blood, joints, skin, and soft tissue. The blood infection rate of *S. lugdunensis* is around 0.3% [9], which is lower than most other bacteria. However, there are an increasing number of reports on blood infections caused by this bacterium [10,11].

The prevalence of *S. lugdunensis* varies greatly among different geographical regions, including 1.3% in Japan [12], 0.8% in Korea [13], 3% in the U.S. [14], and 6% in Argentina [15]. While it is suspected that the incidence of this bacterium in Asiatic countries is similar, its incidence has not yet been investigated in China. One reason for the low detection and underappreciated infection rates of *S. lugdunensis* are that most clinical microbiology laboratories do not usually speciate CoNS [7,16]. Therefore, accurate methods are needed in order to accurately determine incidence by speciation of CoNS isolates. While Frank et al. suggested that ornithine decarboxylase (ODC) and pyrrolidonyl arylamidase (PYR) tests could identify *S. lugdunensis* from CoNS [17], Tan et al. showed that these two tests could only be used as a preliminarily screen for the bacterium [18]. Currently, it is believed that the sequence of the glyceraldehyde-3-phosphate dehydrogenase-encoding (gap) gene can be used to accurately identify *S. lugdunensis*[19]. Additionally, the current problem of drug resistance in CoNS isolates is severe [20]. The rate of drug resistance of *S. lugdunensis* varies throughout the world and while it is susceptible to most antibiotics, there are case reports on its resistance to some drugs [17,18,21,22].

The objectives of the present study were to determine the frequency of *S. lugdunensis* in 670 non-replicate CoNS clinical isolates from the General Hospital of the People’s Liberation Army in China and to clinically and microbiologically characterize them. Specifically, we determined drug resistance patterns and molecular epidemiological characteristics, contributing to the clinical diagnosis and treatment of *S. lugdunensis* infections.

## Results

### Detection of *S. lugdunensis* isolates

**Figure 1:**
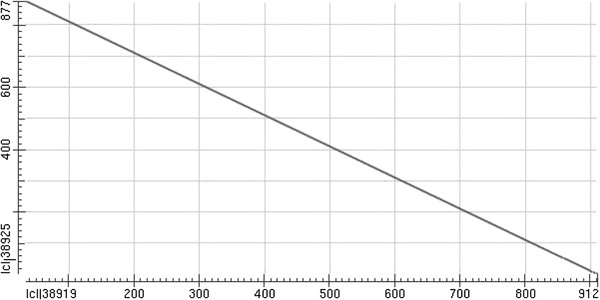
**Dot matrix view of the BLAST results showing regions of similarity of the five isolates.** The query sequence is represented on the X-axis and the numbers represent the bases/residues of the query. The subjects are represented on the Y-axis and again the numbers represent the bases/residues of the subject. Alignments are shown in the plot as lines. Minus strand matches are slanted from the upper left to the lower right. The number of lines (n = 1) shown in the plot is the same as the number of alignments (n = 1) found by BLAST. Query coverage was 96% and maximum identity was 99%.

Eight out of the 670 isolates were positive for both ODC and PYR (single positives were not pursued further). Isolate 2 and 4 were positive in the Latex Agglutination test; however, only Isolate 4 was positive in the Slide Coagulase test. All isolates were negative in the subsequent Tube Coagulase test. Of these eight isolates, 4 were further validated by both VITEK 2 GP and API 20 Staph, with a sensitivity of 80% (4/5), one could not be accurately identified by either, and the other 3 were identified as *S. haemolyticus* (Table 1). The sequences of the *gap* gene for all 5 isolates were 99% identical to the corresponding *S. lugdunensis* sequence (GenBank accession number AF495494.1) (Figure 1). Hence, five out of the 670 CNS isolates were detected as being *S. lugdunensis*, a detection rate of 0.7% (5/670). Of the of five *S. lugdunensis* isolates identified, one was obtained from the venous blood of a patient with influenza, one was from the synovial fluid of a patient with a joint infection, three were from secretions (mammary, cervical, and wound secretions) of patients with skin and soft tissue infections (Table 2).

**Table 1:** **Identification results of API 20 Staph, VITEK 2 GP,*gap*gene sequencing, tube coagulase, slide coagulase, and latex agglutination tests**

**No.**	**API 20 Staph**	**VITEK 2 GP**	**Gap gene**	**Tube Coagulase**	**Slide Coagulase**	**Latex Agglutination**
	**(Identification rate**^**1**^**)**	**(Identification rate**^**1**^**)**	**(Similarity**^**2**^**)**			
**1**	*S. hominis* (73%)	*S. hominis* (50%)	*S. lugdunensis* (99%)	-ve	-ve	-ve
**2**	*S. lugdunensis* (90%)	*S. lugdunensis* (94%)	*S. lugdunensis* (99%)	-ve	-ve	**Positive**
**3**	*S. haemolyticus* (96%)	*S. haemolyticus* (99%)	*S. haemolyticus* (99%)	-ve	-ve	-ve
**4**	*S. lugdunensis* (85%)	*S. lugdunensis* (99%)	*S. lugdunensis* (99%)	-ve	**Positive**	**Positive**
**5**	*S. haemolyticus* (53%)	*S. haemolyticus* (94%)	*S. haemolyticus* (100%)	-ve	-ve	-ve
**6**	*S. lugdunensis* (94%)	*S. lugdunensis* (99%)	*S. lugdunensis* (100%)	-ve	-ve	-ve
**7**	*S. haemolyticus* (92%)	*S. haemolyticus* (99%)	*S. haemolyticus* (99%)	-ve	-ve	-ve
**8**	*S. lugdunensis* (94%)	*S. lugdunensis* (99%)	*S. lugdunensis* (99%)	-ve	-ve	-ve

-ve in Latex Agglutination test signifies no noticeable clearance of the blue background in the latex test; -ve in Slide Coagulase test signifies no visible clumping or clotting using either saline or plasma; -ve in Tube Coagulase test signifies no clot by the end of 4 hours or following 24 hours incubation a room temperature.

^1^The highest percentage of identification.

^2^The highest similarity after aligning by BLAST.

**Table 2:** **Clinical characteristics of*S. lugdunensis*isolates**

**ID**	**Isolate No.**^**1**^	**Department**	**Age (years old)/Gender**	**Diagnosis**	**Fever**	**Leukocyte increase**	**Specimen resource**	**C-reactive protein (mg/dl)**	**Results**
1	1010-13169	Outpatient Clinic	48, female	Mammitis	No	No	Secretion	Unavailable	Heal
2	1010-13159	Orthopedics	69, male	10 years after right knee joint replacement	Yes	No	Synovial fluid	4.8	Heal
4	1001-17088	Obstetrics	37, female	Premature rupture of fetal membranes, gestational diabetes	Yes	Yes	Cervical secretion	3.6	Heal
6	1012-23199	Orthopedics	56, female	Infection after left tibial plateau fracture surgery	Yes	No	Wound secretion	7.50	Heal
8	1002-04128	Neonate^2^	0, male	Neonatal pneumonia and septicemia	Yes	No	Venous blood	0.1	Heal

^1^Isolate No. in the General Hospital of the People’s Liberation Army; ^2^The patient was a newborn of 14 days.

### Characterization of drug resistance in the *S. lugdunensis* isolates

**Figure 2:**
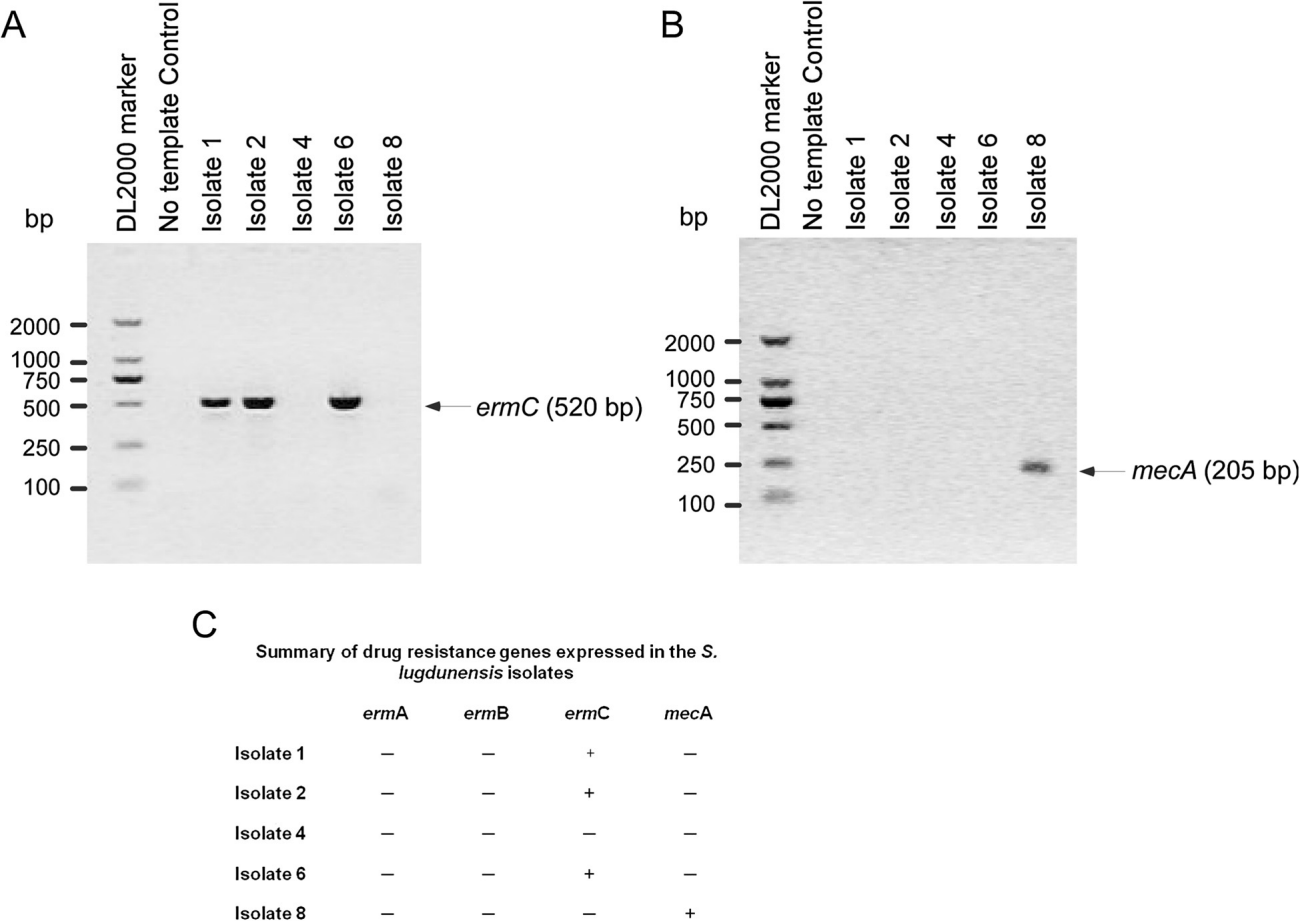
**Gel Electrophoresis of PCR amplification products of resistance genes, *erm *A (A), and *mec *A (B) in the five positive and confirmed isolates (Isolates 1, 2, 4, 6, and 8) of*Staphylococcus lugdunensis. *** Whereas *erm *A was amplified for 35 cycles, *mec *A was amplified for 30 cycles.

Kirby-Bauer (K-B) disc diffusion tests showed that among the five isolates of *S. lugdunensis*, three were resistant to erythromycin (ERM), clindamycin (DA), and penicillin (P), one was resistant to cefoxitin and penicillin and positive for β-lactamase, and one was susceptible to all antimicrobials and negative for β-lactamase (Table 3). E-TEST results indicated that the 5 isolates were susceptible to vancomycin (VA) (Table 3). Results for control strains for both methods were within the reference ranges. The *ermC* resistance gene was present in 3 of the 5 isolates of *S. lugdunensis*, as determined by PCR amplification (Figure 2A). None of the isolates had *erm*A or *erm*B genes (data not shown), whereas the *mecA* gene was present in one isolate (Figure 2B). The PCR results are summarized in Figure 2C.

**Table 3:** Results of drug susceptibility test assayed by the Kirby-Bauer and E-Test and β-lactamase assay

**ID**	**SA**^**1**^	**CFZ**^**1**^	**E**^**1**^	**FOS**^**1**^	**FOX**^**1**^	**GM**^**1**^	**DA**^**1**^	**LVX**^1^	**LZD**^**1**^	**P**^**1**^	**RA**^**1**^	**CXM**^**1**^	**SXT**^**1**^	**VA**^**2**^	**β-lactamase**
1	27	34	6(R)^*^	30	30	26	18(R)^*^	29	34	15(R)^*^	32	34	28	1.2	+
2	28	34	6(R)^*^	30	30	28	6(R) ^*^	26	32	14(R)^*^	34	32	26	1.0	+
4	40	44	36	46	28	30	36	28	36	40	40	40	32	1.5	-
6	20	38	6(R)^*^	26	35	26	6(R) ^*^	29	34	9(R) ^*^	38	40	26	1.0	+
8	21	24	32	26	18(R)^*^	27	34	26	34	14(R)^*^	40	23	32	0.8	+

^1^Inhibition zone (mm); ^2^Minimum inhibition concentration (MIC) (μg/ml); *Drug resistant (*R*).

*ID* identification directory, *SA* ampicillin/sulbactam, *CFZ* cefazolin, ERM erythromycin, *FOS* fosfomycin, *FOX*: cefoxitin, *GM* gentamicin, *DA* clindamycin, *LVX* levofloxacin, *LZD* linezolid, *P* penicillin, *RA* rifampicin, *CXM* cefuroxime, *SXT* trimethoprim + sulfamethoxazole, *VA* vancomycin).

### PFGE did not reveal widespread diversity among the isolates

**Figure 3:**
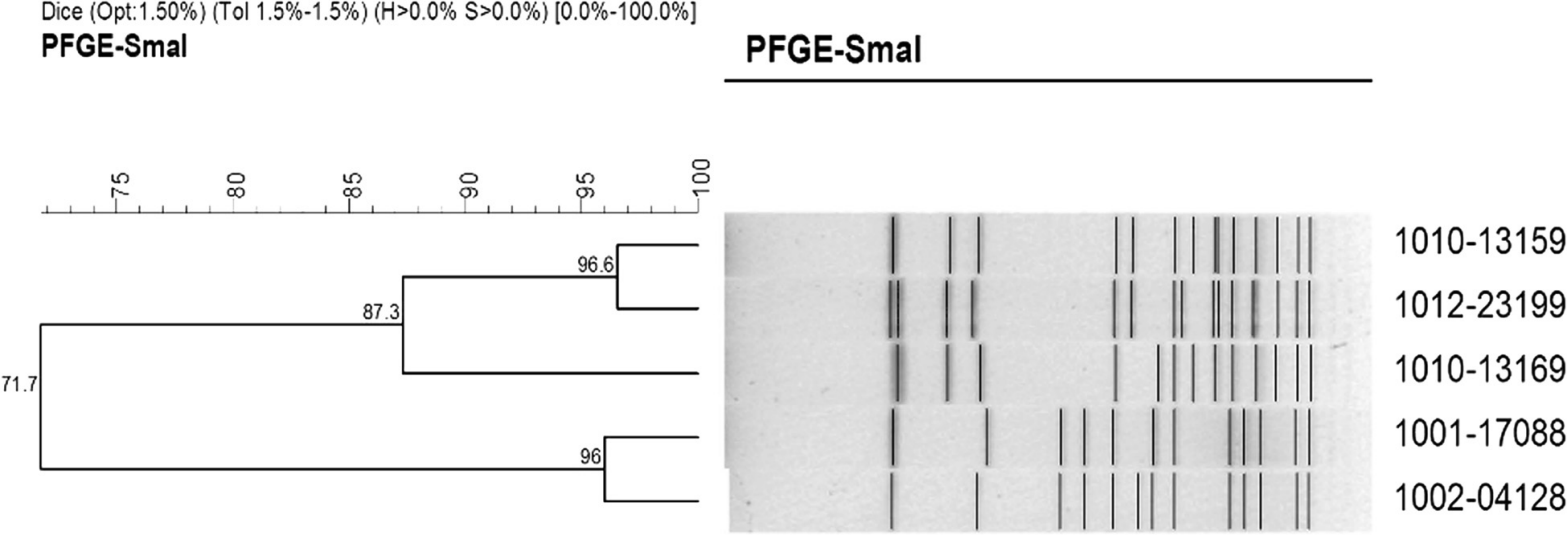
**Cluster dendrogram of *Sma *I pulsed-field gel electrophoresis patterns of the five positive and confirmed *S. lugdunensis *isolates.** Colonies of each isolate were lysed using lysostaphin and DNA was subsequently digested with *Sma*I. Pulsed-field gel electrophoresis (PFGE) was performed using the CHEF-DR III system on a 1% agarose in 0.5 X TBE buffer for a run time of 18 h, with a voltage of 6 V/cm, pulses ramped from 4.0 to 40.0 s, at an angle of 120°. Gels were stained with ethidium bromide and photographed using a Gel Doc 2000 before band patterns were analyzed with BioNumerics version 3.0.

After *Sma*I digestion and electrophoresis, genomic DNA fragments were well separated and 12 to 15 DNA electrophoretic bands were produced (Figure 3). A cluster dendrogram did not reveal widespread diversity, with similarity among the five isolates ranging from 71.7% to 96.6%; two pairs of isolates were 96.0% and 96.6% similar and one isolate had below 87.3% similarity to the other isolates (Figure 3).

## Discussion

To the best of our knowledge, this is the first report of prevalence of *S. lugdunensis* in clinical specimens obtained from mainland China. An earlier case of *S. lugdunensis* was reported in a 31-year-old Chinese patient (suffering from right ear sinus) in Singapore in 2000 [23]. Recently, community acquired *S. lugdunensis* were reported in clinical infections associated with co-morbidities in Southern Taiwan [24]; however the study did not observe widespread antimicrobial resistance even though there was widespread genetic diversity in the confirmed isolates. In the current study, our detection rate was 0.7% (5/670), which is comparatively on the lower end [12,14,15], but similar to what has been reported in Korea [13].

In the revised manuscript, we have described in details (in the legend of Table 1) what –ve (negative) signifies for the tube coagulase, slide coagulase, and latex agglutination test, respectively.

The latex agglutination test and slide coagulase test are used for rapid identification or for ruling out *Staphylococcus aureus*. However, some *Staphylococcus* isolates produce a membrane-bound form of the clumping factor, which can yield a positive result in slide coagulase and/or rapid latex agglutination tests, thus requiring the confirmatory tube coagulase test. However, an isolate that is positive in the Latex Agglutination test has a high probability of a positive slide coagulase test and our assay for Isolate 2 does not conform to this, whereas Isolate 4 does. In addition, recent results have shown that the prevalence of the fibrinogen-binding adhesion (*fbl*) is 100% in *Staphylococcus lugdunensis* isolates [25]. However, one recent study reported that of the 17 *Staphylococcus lugdunensis* isolates studied, though *fbl* gene could be detected in all cases, only 47% gave a positive slide coagulase test result [26]. This perhaps suggests that varying levels of *fbl* gene product dictates the apparent sensitivity of *Staphylococcus lugdunensis* isolates to slide coagulase test. On comparing the results for Isolate 2 and 4 (both positive for latex agglutination test, but only Isolate 4 positive in slide coagulase test), they differ markedly in drug resistance and β-lactamase expression, with Isolate 4 being susceptible to all drugs tested and the only isolate not expressing β-lactamase. It is difficult to say whether the *fbl* gene expression pattern dictates this apparent difference between these isolates; however, it will be very interesting to see in the future if there is any difference in sensitivity to latex agglutination and slide coagulase test based on *fbl* gene expression in *Staphylococcus lugdunensis* isolates.

We used PYR and ODC tests to preliminarily screen CNS isolates, VITEK 2 GP and API 20 Staph to validate, and *gap* gene sequence to confirm *S. lugdunensis* isolates. There are 27 CoNS species registered in GenBank (Accession numbers DQ321674 to DQ321700). The amplification of the partial *gap* gene for all of the *Staphylococcus* species (sequence similarity ranges from 24.3 to 96%) yield a single product of nearly 931 bp [19]. The sequence similarity of the *gap* sequences ranged from 24.3 to 96% [19]. In fact, in our analyses the second closest strain was *S. haemolyticus* (data not shown), which has a *gap* gene sequence similarity of 27% [19] with *S. lugdunensis*[19]. We found that among the 8 isolates positive in both PYR and ODC tests, 5 were *S. lugdunensis* and the other three were *S. haemolyticus*. This may due to *S. haemolyticus* being weakly positive for ODC, which is consistent with previous results [27].

Of the 5 isolates of *S. lugdunensis* identified in this study, 3 were obtained from wound, breast, and cervix secretions, suggesting that skin and soft tissue infections account for a prominent number of the total infections caused by *S. lugdunensis*, which is consistent with previous results [17]. One isolate was from the synovial fluid of the patient with a joint infection. Frank et al. reported that this bacterium infects artificially replaced joints [28] and it accounts for 4% of all joint infections [29]. Another isolate was from the venous blood of a newborn baby with pneumonia. Tee *et al.* previously reported a case of neonatal pneumonia caused by this bacterium but that case suffered from a catheter-related blood infection [8]. Consistent with previous results [13], *S. lugdunensis* was not isolated from any sputum cultures in this study, which may be due to inability of this bacterium to colonize the respiratory tract.

Four out of the five *S. lugdunensis* isolates identified in this study produced β-lactamase (Table 3), which indicates an incidence of 80% that is much higher than the incidence in other countries [17], including 7-24% in France, 24-40% in the U.S., 12% in Spain, and 15% in Sweden. Of note, our small number of positive isolates might have potentially biased such estimations. Only one out of the five isolates was not resistant to the antimicrobial drugs tested, three were resistant to erythromycin, clindamycin, and penicillin, and one was resistant to cefoxitin and penicillin (Table 3). We found that the three isolates resistant to erythromycin were positive for the *ermC* gene but not the *ermA* or *ermB* gene; and the isolate resistant to cefoxitin was positive for the *mecA* gene; the later was only reported a few times in the previous studies [8,30,31]. We further found that the rate of antibiotic resistance of *S. lugdunensis* is more severe in China than in other countries and primarily presented as multi-drug resistance, again such an inference might suffer from potential bias due to the sample size of the confirmed isolates.

We performed PFGE in order to determine the epidemiological characteristics of *S. lugdunensis* in our hospital, even though the sample size was not ideal for accurately defining epidemiological characteristics. Of note, *Sma*I-restricted *S. lugdunensis* in order to gain a band pattern is known to be more difficult compared to *S. aureus* due to methylation of *Sma*I sites [32]. These isolates were not typed due to the small sample size. However, a cluster dendrogram and clinical analysis still provide epidemiological characteristics. The two isolates with a similarity of 96.0% were from a patient with a premature rupture of fetal membranes and a 14-day-old newborn. The isolate with a similarity of 87.3% or less with other isolates was from the outpatient clinic. The two isolates with a similarity of 96.6% were from the Department of Orthopedics, were both resistant to erythromycin, clindamycin, and penicillin and produce β-lactamase, suggesting that PFGE can provide epidemiological information for *S. lugdunensis* from different departments.

## Conclusions

In summary, while the prevalence of *S. lugdunensis* in our study is low and warrants further investigations, it is of significant clinical concern that its rate of multi-drug resistance is so high. The diversity of *S. lugdunensis* by macrorestriction analysis with *Sma*I was limited for typing (due to sample size) but sufficient to consider that PFGE with *Sma*I is suitable for epidemiological analyses. Further studies encompassing detailed molecular methods similar to the current one will be required to characterize the nationwide prevalence and genetic diversity of the β-lactamase positive *S. lugdunensis* isolated in China.

## Methods

### Collection of bacterial isolates

The Institutional Scientific and Ethics Committees of the General Hospital of the People’s Liberation Army approved the current study. Between January and December of 2010, 670 non-replicate isolates of CoNS were collected from clinical specimens in our hospital, inclusive of blood (n = 74), sputa (n = 188), secretions (n = 84), synovial fluid (n = 17), semen (n = 19), drainage fluid (n = 52), pus (n = 52), nose swabs (n = 20), throat swabs (n = 36), urine (n = 116), catheters (n = 13), and others (n = 36). All isolates were obtained after informed consent of the patients. The isolates were all stored at −86°C.

### DNA extraction

Bacterial colonies cultured overnight on blood agar plates were suspended in 2 ml 0.85% NaCl solution to 5 McFarland units and centrifuged at 13,000 g for 1 min. The pellets were resuspended in 200 μL lysis buffer solution [1% Triton X-100, 10 mM Tris–HCl (pH 8.0), and 1 mM EDTA], boiled for 10 min, and centrifuged at 13,000 g for 2 min. Supernatants were collected and stored at −20°C.

### Identification of *S. lugdunensis*

*S. lugdunensis* was isolated and identified from CoNS in three steps. First, the 670 isolates were screened successively with ornithine decarboxylase (ODC) and pyrrolidonyl arylamidase (PYR), and those that were positive for both (n = 8) were considered as suspected isolates of *S. lugdunensis*. Second, the suspected isolates were further validated with API 20 Staph and VITEK 2 gram-positive (GP) kits (bioMérieux, France). Finally, the *gap* gene of the identified *S. lugdunensis* isolates was sequenced as the confirmatory detection tool. The following primers were used to amplify 933 bp of the *gap* gene [19]: 5′-ATGGTTTTGGTAGAATTGGTCGTTTA-3′ (forward) and 5′-GACATTTCGTTATCATACCAAGCTG-3′ (reverse). The PCR reaction was performed in a volume of 25 μL with 2.5 μL of 10× PCR Buffer (Mg^2+^ Plus), 2 μL of 2.5 mM dNTPs, 1 μL of 10 μM primers, 0.025 U Taq DNA polymerase (TaKaRa), 15.5 μL of double distilled water (DDW), and 4 μL of target DNA. The amplification was performed using a Veriti Thermal Cycler (Applied Biosystems, Foster City, CA) with an initial denaturation at 94°C for 2 min, 40 cycles of denaturation at 94°C for 20 s,annealing at 55°C for 30 s, elongation at 72°C for 40 s, and a final elongation at 72°C for 5 min. The sequences were aligned to the *S. lugdunensis* sequence (GenBank accession number AF495494.1) using the BLASTN 2.2.26+ program [33]. Isolates were confirmed to be *S. lugdunensis* if the sequence similarity was greater than 99%.

### Detection of antimicrobial susceptibility and resistance genes

β-lactamase was detected with the rapid detection kit (bioMérieux, France) using *Staphylococcus aureus* ATCC 29213 as positive control strain and *Enterococcus faecalis* (ATCC 29212) as a negative control strain. Drug susceptibility tests were performed and interpreted following M100-S20 standards set by the Clinical Laboratory Standards Institute (CLSI) in 2010 [34]. Susceptibility to vancomycin (VA), ampicillin/sulbactam (SA), cefazolin (CFZ), erythromycin (ERM), fosfomycin (FOS), cefoxitin (FOX), gentamicin (GM), clindamycin (DA), levofloxacin (LVX), linezolid (LZD), penicillin (P), rifampicin (RA), cefuroxime (CXM), and trimethoprim + sulfamethoxazole (SXT) was tested with the E-TEST and K-B methods using ATCC29213 and ATCC 25923 as control strains, respectively.

*S. lugdunensis* isolates were tested for the antibiotic resistance genes *ermAermBermC* (erythromycin resistance), and *mecA* (cefoxitin resistance) using primer sequence and conditions described before [35-37]. Briefly, the *ermA* and *ermC* genes were amplified with an initial denaturation at 95°C for 5 min, followed by 35 cycles of denaturation at 95°C for 50 s, annealing at 52°C for 45 s, elongation at 72°C for 50 s, and a final elongation at 72°C for 7 min. The parameters for PCR amplification of *ermB* were an initial denaturation at 95°C for 5 min, then 35 cycles of denaturation at 94°C for 50 s, annealing at 55°C for 50 s, elongation at 72°C for 1 min, and a final elongation at 72°C for 7 min. Amplification parameters for the *mecA* gene were an initial denaturation at 95°C for 5 min, then 30 cycles of denaturation at 95°C for 30 s, annealing at 50°C for 20 s, elongation at 72°C for 20 s, and a final elongation at 72°C for 5 min.

### Pulsed-Field Gel Electrophoresis (PFGE)

Colonies of each isolate were suspended in 2 ml cell suspension buffer such that they read 4.5 (colorimeter standard, BioMérieux Vitek) on a McFarland Colorimeter. Bacterial cells were lysed using 2 μL lysostaphin (1 mg/mL, Sigma) in a 250 μL bacterial suspension and DNA was digested with *Sma*I (TaKaRa). Pulsed-field gel electrophoresis (PFGE) was performed using the CHEF-DR III system (Bio-Rad) on a 1% agarose (Cambraex Bio Science, Rockland) in 0.5 X TBE buffer (45 mM Tris-borate, 1 mM EDTA) for a run time of 18 h, with a voltage of 6 V/cm, pulses ramped from 4.0 to 40.0 s, at an angle of 120°. The standard strain H9812 (*Xba*I enzyme) was used as the electrophoresis marker. Gels were stained with 1 μg/mL ethidium bromide for 30 min, washed in water for 30 min, and photographed using a Gel Doc 2000 (Bio-Rad). Band patterns were analyzed with BioNumerics version 3.0 (Applied Maths BVBA, Belgium) with the Dice coefficient and UPGMA clustering at 1.5% band tolerance.

## Competing interests

The authors declare that they do not have any competing interest.

## Authors’ contributions

C.L. and S.D. designed the experimental plan. C.L. performed most of the experiments; G.J. and W.K. did strain collection and isolation, respectively; W.H. did *gap* gene sequencing analysis; Y.Z. performed PFGE data analysis; C.R. participated in strain identification Y.L. performed drug resistance phenotype detection; C.L. and S.D. analyzed the data and wrote the manuscript; all authors have reviewed the manuscript.
